# A case of trichotillomania with binge eating disorder: combined with N-acetylcysteine synergistic therapy

**DOI:** 10.1186/s12991-021-00369-9

**Published:** 2021-09-25

**Authors:** Xudong Zhao, Shikai Wang, Xiujuan Hong, Shaojia Lu, Sufang Tang, Yue Shen, Ming Feng, Ping Guo, Yu Fang

**Affiliations:** 1 Department of Psychiatry, Huzhou Third Municipal Hospital, The Affiliated Hospital of Huzhou University, 2088 Tiaoxi Road, Huzhou, 313000 Zhejiang China; 2 Department of Psychiatry, The First Affiliated Hospital, Zhejiang University School of Medicine, Hangzhou, China

**Keywords:** N-Acetylcysteine, Trichotillomania, Binge eating disorder

## Abstract

**Background:**

Obsessive–compulsive and related disorders (OCRDs) are a group of intractable and chronic mental disorders. Trichotillomania (TTM) is a common type of OCRDs characterized by repetitive hair pulling, driven by escalating tension before the action and during the attempts to resist it. Binge eating disorder (BED) is a common type of eating disorder characterized by recurrent compulsive episodes of binge eating. Both have common psychological processes (tension or impulsion) and pathological manifestations (out of control), but the pathological mechanisms are still unclear and the current clinical treatments are often unsatisfactory for these two disorders.

**Case presentation:**

A 25-year-old woman with TTM comorbid BED came to our hospital for treatment. She had accepted systematic cognitive behavioral therapy (CBT) and also monotherapy or multidrug therapy with sertraline, fluvoxamine, bupropion, risperidone in full dosage and duration for 2 years, but all of them did not work. We treated this case with N-acetylcysteine (NAC) as a synergist on the basis of recent treatment (fluvoxamine 150 mg/day and bupropion 300 mg/day). The pathological hair plucking behavior and binge eating symptoms were both significantly and rapidly improved, and the follow-up in next 14 weeks showed that the effect was still maintained.

**Conclusion:**

To our knowledge, this may be the first case report of using NAC as a synergist to treat TTM comorbid BED successfully, which suggest that these two disorders may have a common pathophysiological mechanism. Moreover, NAC can be one choice as a synergistic treatment for OCRDs.

## Background

Obsessive–compulsive and related disorders (OCRDs) are a group of intractable and chronic mental disorders in which the pathological mechanism is still unclear and in which the therapeutic effect is unsatisfactory [1]. Additionally, these disorders are independent chapters in the 5th edition of the Diagnostic and Statistical Manual of Mental Disorders (DSM-V). Therefore, the mainstream anti-obsessive–compulsive treatment is used with selective serotonin reuptake inhibitors (SSRIs) and cognitive behavioral therapy (CBT) as the first-line treatment. However, regardless of whether SSRIs and CBT were used alone or in combination, the effective rate was only approximately 40–60% [2], other reports showed that only 20% of patients are cured [3]. Previous studies showed that the effect sizes of OCD treatment were 0.37–1.09 for drug treatment and 0.99–1.13 for CBT. Even when synergistic therapy with recommended drugs such as aripiprazole, the efficacy remains limited, which results in great pain and burden to patients and their families. According to the medication procedures for obsessive–compulsive disorder of the Harvard Southbank Program, when there are both enough quantity and enough treatment with first-line drugs and conventional synergistic regimens that have poor efficacy in patients with OCD, glutamate modulators, including NAC, can be selected for use as a synergistic therapy [4]. Recent genetic and pharmacological studies have particularly highlighted glutamate dysregulation as a possible contributor to OCD, and several glutamate modulators have shown promise in early studies [5]. One study summarizes investigations of memantine, riluzole, ketamine, D-cycloserine, glycine, NAC, topiramate, and lamotrigine. Evidence exists for benefit from each of these in some patients; these agents are options in individuals whose symptoms are refractory to better-established therapeutic strategies [6].

Trichotillomania (TTM) is a common type of OCRDs characterized by repetitive hair pulling, driven by escalating tension before the action and during the attempts to resist it, and causing variable hair loss. Currently, it lacks specific drugs for treatment, the current standard of treatment is still SSRIs, supplemented by psychological and behavioral therapy.

Binge eating disorder (BED) is characterized by recurrent compulsive episodes of binge eating, as previously defined, accompanied by a sense of lack of control and also by eating more rapidly than normal; until feeling uncomfortably full; without being hungry; having disgust emotion for oneself and with marked distress after the occurrence of the episodes [7]. There is still a lack of definitive treatment for BED. A meta-analysis of BED show effectiveness up to 12 months following treatment was demonstrated for psychotherapy, structured self-help treatment, and combined treatment, while the results regarding body weight reduction were inconsistent [8].

Both of TTM and BED have common psychological processes (tension or impulsion) and pathological manifestations (out of control), but the pathological mechanisms are still unclear and the current clinical treatments are often unsatisfactory for these two disorders. Here are few studies on the diagnosis and treatment of their comorbidity.

Herein, we describe a female TTM comorbid BED patient who exhibited fast and dramatic improvements in her hair pulling and binge eating symptoms after combined NAC synergistic treatment when conventional anti-obsessive therapy was ineffective. This case is helpful for understanding the pathogenesis of TTM and BED, and also enriches our clinical experience for the application of NAC in OCRDs and obsessive–compulsive spectrum disorders (OCSDs).

## Case presentation

Mrs A, a 25-year-old woman, came to our hospital because of repeated hair pulling and binge eating behavior voluntarily. Her medical history was collected via a detailed inquiry. More than 10 years ago, the patient was sexually discriminated against by her family and had a bad relationship with her parents. She gradually became unhappy, self-abased and nervous, and began to pull out her hair repeatedly and uncontrollably, which was more obvious when she was in a bad mood. After about 2 years of systematic CBT and pharmacotherapy, she still had no improvement. Nearly a year ago, the condition worsened; she was upset, unhappy and was crying often. Additionally, the hair pulling phenomenon worsened, and she even used nail clippers to pull out the hair follicles on her head, and she had to resort to constantly wearing a wig to cover up the local hair loss. Furthermore, patients encountering increased pressure will exhibit intermittent binge eating attacks: she will eat much more food than usual in a short time, felt unable to control eating. When this began, she will eat much faster than normal, and until she felt uncomfortably full and got a feeling of guilty after eating, about two to three times a week, but no emetic, diuresis or diarrhea behavior and resulted in a significant increase in weight gain. The patient had an obvious impairment in social function and had changed jobs several times in the last year. Mrs A had been misdiagnosed with "obsessive–compulsive disorder", "depression" or "anxiety disorder" and had been mono- or co-treated with sertraline (200 mg/day × 8 weeks), fluvoxamine (150 mg/day × 12 weeks), bupropion (300 mg/day × 6 weeks) and risperidone (1 mg × 8 weeks) in the last 2 years, but all of them did not work. The patient was given fluvoxamine (150 mg/day) and bupropion (300 mg/day) before admission.

**Fig. 1:**
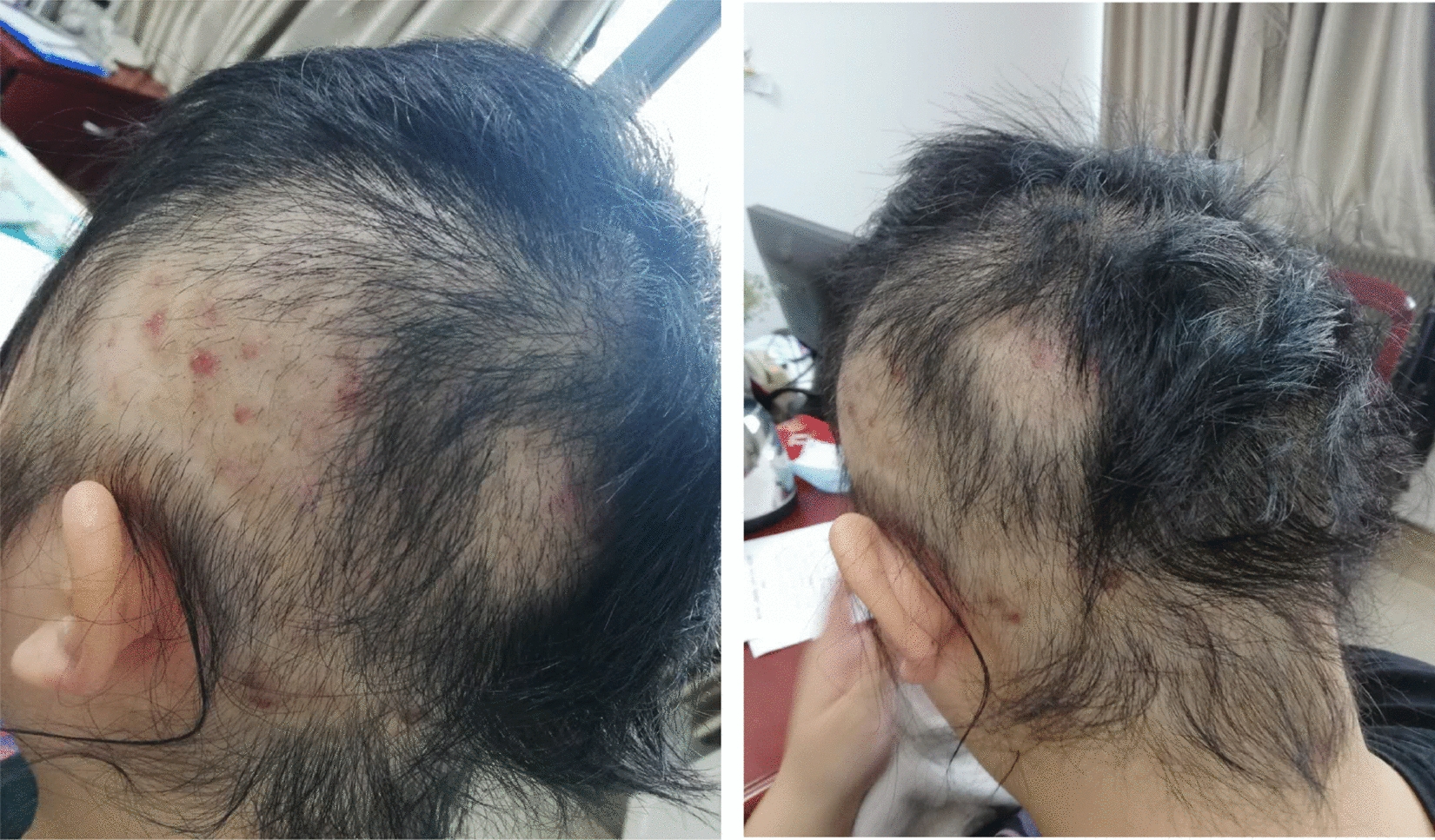
Head photo of the patient before admission for treatment (side and back)

On admission, wearing a wig, Mrs A had normal vital signs, but was obese (BMI: 28.23 kg/m^2^). The hair loss was obvious in the temporal and occipital regions, and the local hair follicles in the scalp were red and swollen with oozed blood (see attached Fig. 1, the local hair defect). Mental state examinations: clear consciousness, normal orientation and cooperation, no delusions or hallucinations, no somatization symptom, she always felt depressed and anxious because she could not control her impulse of pulling hair and binge eating, she had a low self-evaluation but had no suicide attempt, she could not perform well in work and changed her job often. Laboratory tests, ECG, EEG and brain MRI scans showed no obvious abnormalities. The psychological tests revealed: Yale-Brown Obsessive Compulsive Scale(Y-BOCS) showed 18 points, Hamilton Anxiety Scale (HAMA) showed 22 points, Hamilton's Depression Scale-17 (HAMD-17) showed 18 points, Eating Disorder Inventory (EDI) showed 245 points, these results indicate the patient had obvious obsessive–compulsive, depressive and anxiety symptoms, and also indicate she had eating disorder.

After admission on the basis of the recent treatment (fluvoxamine 150 mg/day and bupropion 300 mg/day), the patient was given a combined treatment with NAC, started at 600 mg/day and titrated gradually to 1200 mg/day (D4) and 1800 mg/day (D9). During this period, the patient's anxiety and depression were significantly improved, the hair plucking behavior was significantly reduced and her binge eating was also rapidly alleviated (no recurrence except on the day of admission). Subsequently, the patient was discharged from the hospital on day 11.

**Fig. 2:**
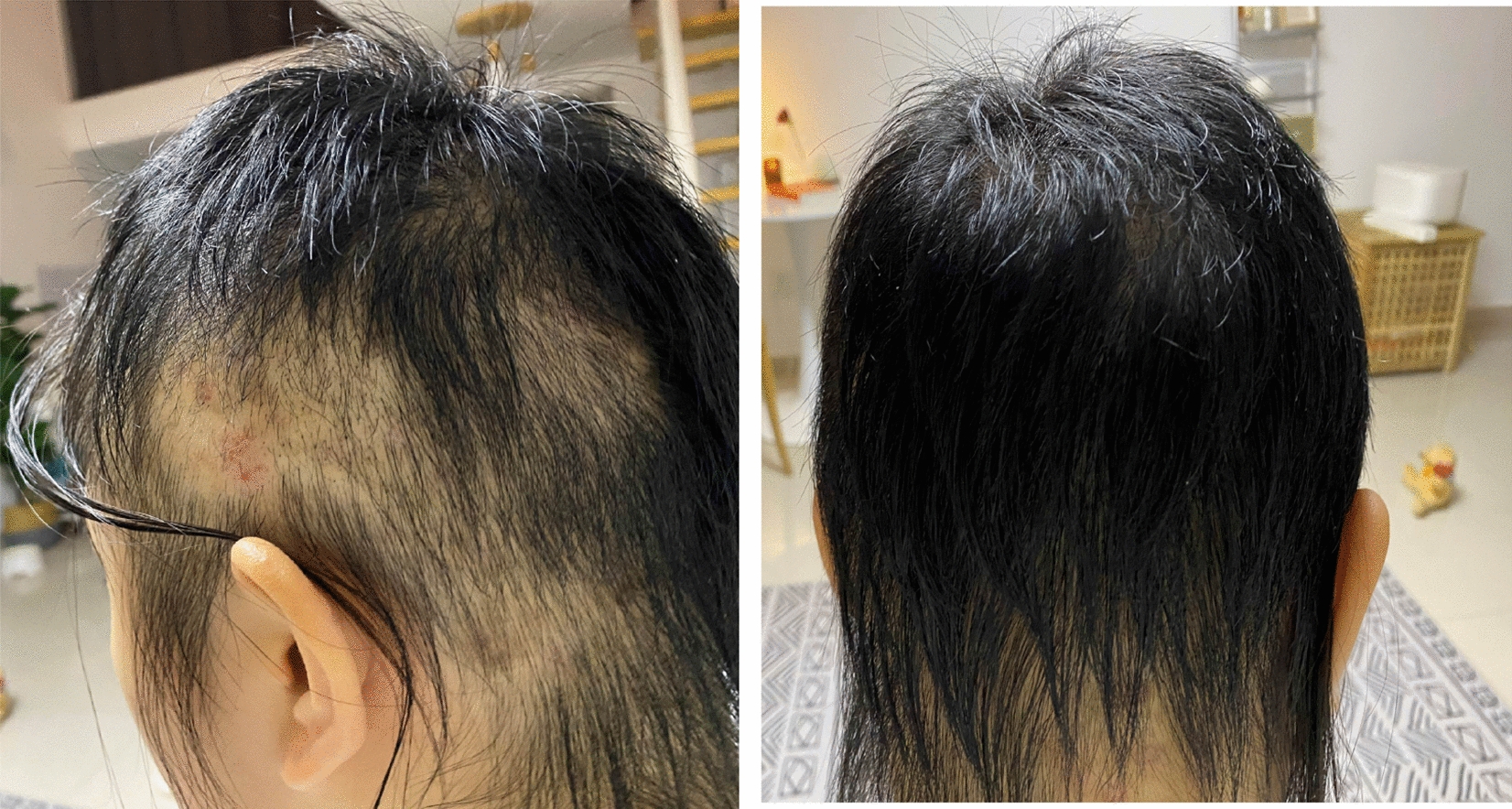
Hair profile at follow-up 2 weeks after discharge (side and back)

**Fig. 3:**
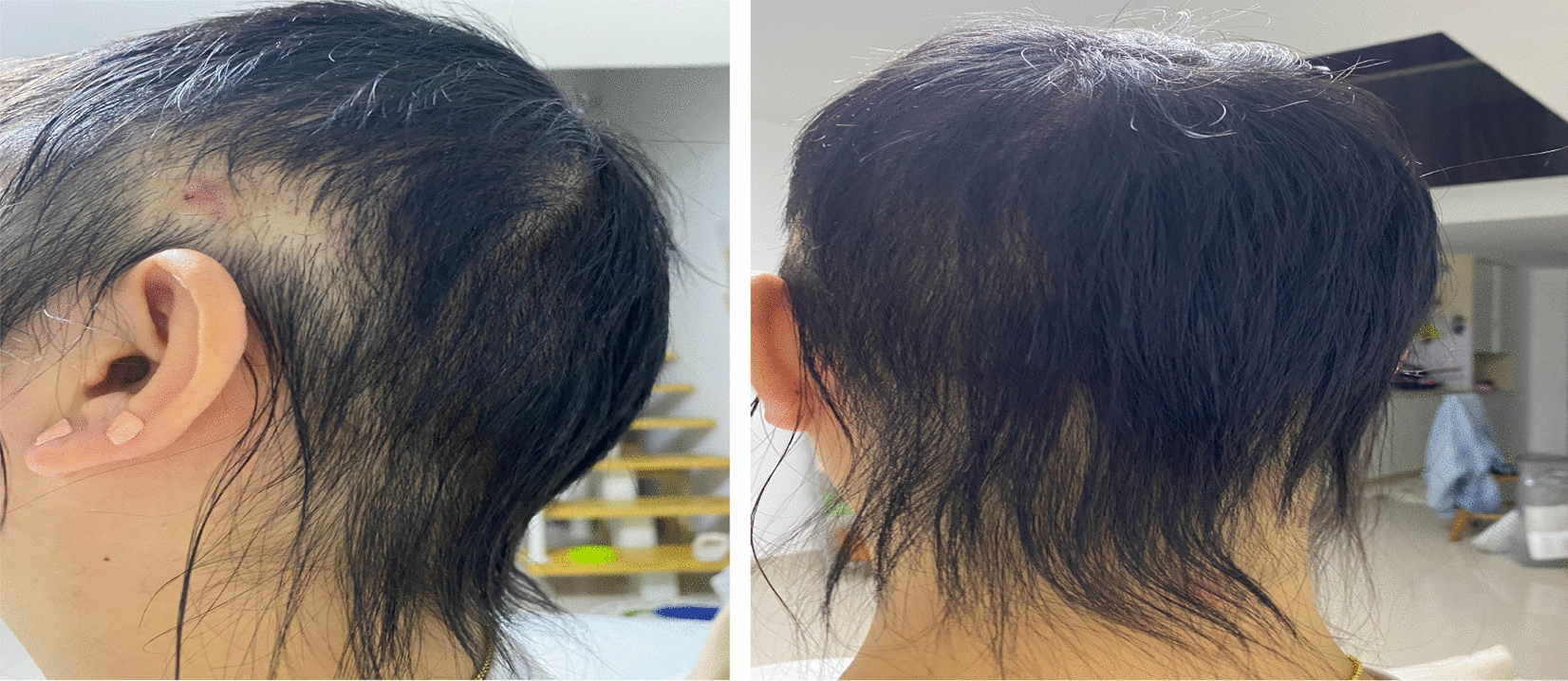
Hair profile at follow-up 4 weeks after discharge (side and back)

**Fig. 4:**
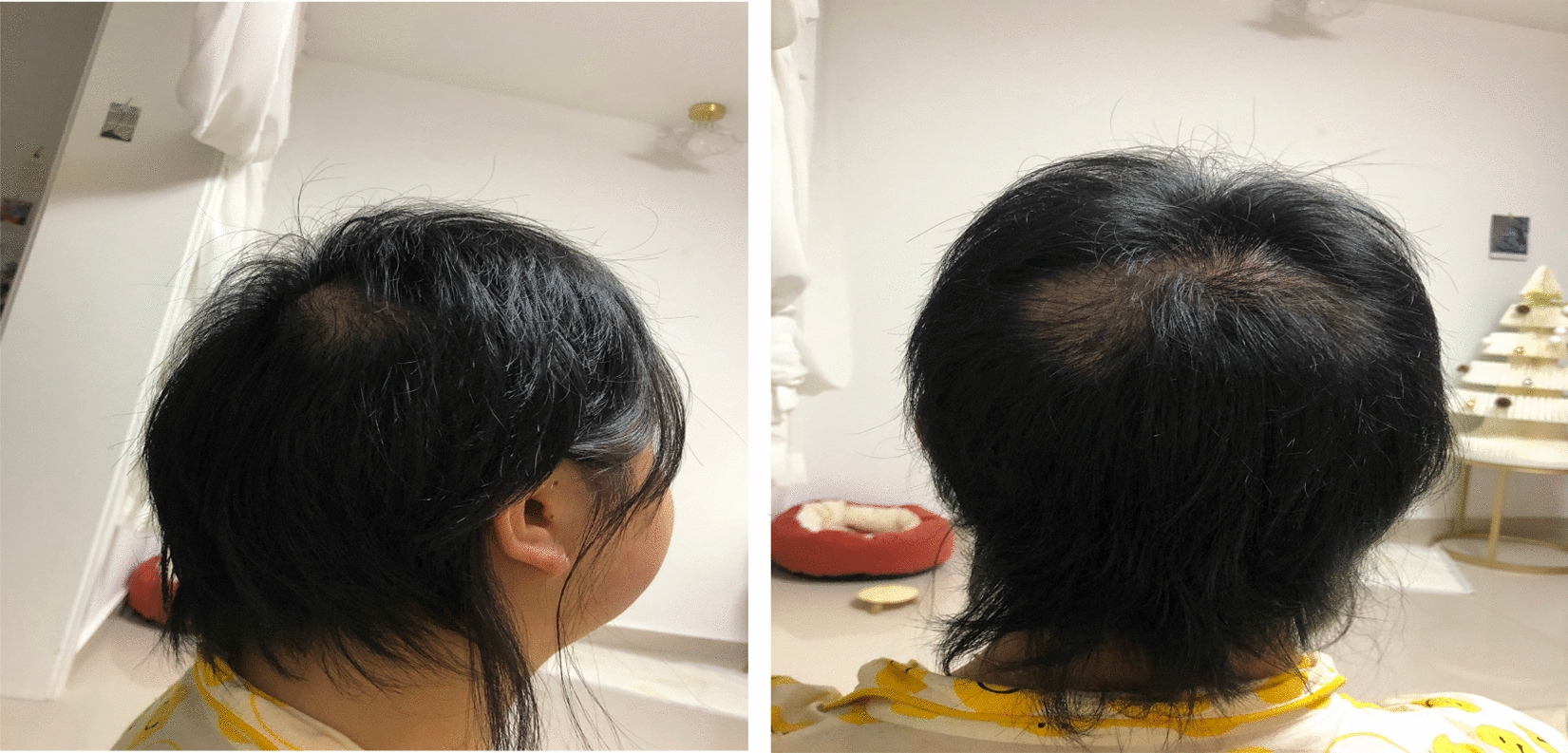
Follow-up of patient's hair 14 weeks after discharge (side and back)

A follow-up of 2 weeks after discharge showed that the patient's mood was stable, her anxiety was alleviated, hair plucking behavior was rarely observed, her hair was thicker than before and inflammation was significantly improved. A follow-up of 4 weeks after discharge showed an improvement of her obsessive symptoms, no episodes of binge eating and her normal occupation of teaching had resumed. A follow-up of 14 weeks after discharge (16 weeks after treatment) showed no plucking behavior, no episodes of binge eating, slimmer than ever (BMI: 26.95 kg/m^2^), and she was in good working and living conditions. Maintenance dose was fluvoxamine 100 mg/day, bupropion 300 mg/day, NAC 1200 mg/day. (drug dosages and scale evaluations used during treatment are shown in Table 1, and changes in local hair defects are shown in Figs. 2, 3 and  4).

**Table 1:** Patients' NAC dosages, Y-BOCs, HAMA-14, HAMD-17, EDI scores

NAC dosages (mg)	Baseline	D1	D3	D4	D7	D9	4 W	6 W
	600			1200		1800		1800
Y-BOCS	18	17	14		11		7	4
HAMA	22	21	17		14		13	8
HAMD	18	16	14		10		9	7
EDI	245	243	221		206		193	183

## Discussion

Impulsivity and obsessiveness are at opposite ends of the spectrum of symptoms, the five categories of impulsivity control disorders in the DSM-IV-TR, though still controversial, have been grouped into different categories in DSM-5 [9]. In addition, there is a growing number of comorbid studies on TTM and eating disorders (ED), both of which are characterized by compulsive behavior [10]. At present, TTM is considered a type of obsessive-related disorder, and BED is classified as an eating disorder [11]. However, previous family studies [12] and cross-sectional surveys [10] suggest that eating disorders are significantly associated with TTM, and the prevalence of TTM in eating disorder patients is much higher than that in the general population [13]. Although the concept of obsessive spectrum disorder has been in existence for a long period of time, there have been disputes about its definition and the category of the disease. After the synergistic treatment of NAC, both the pathological plucking impulsivity and the binge-eating episodes of this patient were rapidly relieved, thus suggesting that both of the conditions may have a common pathophysiological mechanism can and generate the common psychological characteristics of the symptoms [14]. In view of the clinical manifestations and treatment outcomes, both of the disorders can be classified as OCSDs.

NAC is an amino acid used mostly in respiratory medicine to cleave disulfide bonds in sputum, making it easier to cough up. But research in recent years has shown that, it is also a precursor of the inhibitory neurotransmitter γ-aminobutyric acid (GABA), which can regulate the synthesis and secretion of glutamate and dopamine and plays an important role in the process of oxidative stress, apoptosis and neuroinflammation. NAC cysteine compounds (in the reverse adjustment) play an important role in the synthesis of glutamate, with a portion provided by NAC cysteine by the sodium-dependent transport mechanism through the blood–brain barrier, converted into cystine in the brain, and the latter provided via the cystine-glutamate transporter exchange to glutamate, which causes mGLuR2/3 receptor activation and leads to decreased synaptic glutamate release. This restores the extracellular glutamate levels [15], which can be used for the synergistic treatment of obsessive–compulsive disorder. One study showed that NAC is a promising candidate for adjunct treatment for many psychiatric disorders, such as anxiety, bipolar disorder, depression, OCD, OCRD, posttraumatic stress disorder, and schizophrenia [16]. A randomized controlled trial using NAC as an adjuvant with the use of fluoxetine showed significant improvement in the NAC group in the treatment of moderate-to-severe OCD [17].

As a nutritional supplement, NAC is also used as over-the-counter drugs in many countries. At present, NAC has been widely used for respiratory diseases, as well as for detoxification and many types of severe liver damage, and has shown high safety and good tolerance. However, NAC application in the obsessive–compulsive spectrum disorder could be expected by randomized controlled studies with large sample in the future.

## Conclusion

To our knowledge, this may be the first case report of using NAC as a synergist to treat TTM comorbid BED successfully, which suggest that TTM and BED may have a common pathophysiological mechanism, and both of the disorders can be classified as OCSDs. We can try using NAC to treat TTM and BED as a synergistic agent. Moreover, NAC can also be one choice as a synergistic treatment for OCRDs and OCSDs, but further studies are needed to confirm this.

## Data Availability

Not applicable.

## Abbreviations

TTM: Trichotillomania
OCRDs: Obsessive–compulsive and related disorders
BED: Binge eating disorder
OCSDs: Obsessive–compulsive spectrum disorders
NAC: N-Acetylcysteine
DSM: The Diagnostic and Statistical Manual of Mental Disorders
SSRIs: Selective serotonin reuptake inhibitors
CBT: Cognitive behavioral therapy
Y-BOCS: Yale-Brown Obsessive Compulsive Scale
HAMA: Hamilton Anxiety Scale
HAMD-17: Hamilton's Depression Scale-17
EDI: Eating Disorder Inventory
ED: Eating disorders
GABA: γ-Aminobutyric acid
